# Mapping Long-Term Care Needs in Person-Centred Interventions for Older People with Multimorbidity: A WHO Framework-Guided Secondary Analysis

**DOI:** 10.3390/healthcare14121623

**Published:** 2026-06-09

**Authors:** António Lista, Lara Guedes de Pinho, Elisabete Alves, César Fonseca

**Affiliations:** 1 Comprehensive Health Research Centre (CHRC), Universidade de Évora, 7004-516 Évora, Portugal; l.pinho@ess.ipvc.pt (L.G.d.P.); elisabete.alves@uevora.pt (E.A.); cfonseca@uevora.pt (C.F.); 2 Escola Superior de Enfermagem São João de Deus, Universidade de Évora, 7004-516 Évora, Portugal; 3 Escola Superior de Saúde, Instituto Politécnico de Viana do Castelo, 4900-314 Viana do Castelo, Portugal

**Keywords:** long-term care, older people, multimorbidity, person-centred care, integrated care, World Health Organization, framework mapping, psychosocial interventions, rehabilitation

## Abstract

**Highlights:**

**What are the main findings?**

- Person-centred integrated care (18/18 trials), health care needs (17/18), and education and training (17/18) were broadly represented, whereas palliative care needs appeared in only four trials.
- Complementary components were interpreted as supporting the operational infrastructure of care, including assessment, planning, coordination, monitoring, referral, documentation, training, and continuity across intervention packages.

**What are the implications of the main findings?**

- Mapping person-centred interventions to long-term care needs highlights uneven domain coverage and suggests that needs-oriented frameworks add information not captured by technical intervention classifications alone.
- Future trials should explicitly align targeted long-term care needs, delivered components, and outcome selection, including caregiver, palliative, continuity, social, and person-centred care experience outcomes.

**Abstract:**

**Background/Objectives:** Person-centred psychosocial and rehabilitation interventions are increasingly relevant in long-term care (LTC) for older people with multimorbidity. Existing classifications describe the technical nature of these interventions rather than the LTC needs addressed by their delivered components. This study aimed to map delivered or reported components from a published parent review onto the World Health Organization long-term care framework. **Methods:** We conducted a framework-guided secondary analysis of 18 randomised controlled trials, including 9132 participants, from the parent review. Trials were conducted in LTC or settings relevant to LTC. Components were deductively mapped at study level to five framework domains: health care needs, palliative care needs, social care and support needs, person-centred integrated care, and education and training. Mapping followed predefined operational rules, a codebook, and a decision log. Planned-only components were excluded. Results were synthesised descriptively, without reassessing intervention efficacy. **Results:** Health care needs were identified in 17 of 18 trials, social care and support needs in 14, person-centred integrated care in all 18, and education and training in 17. Palliative care needs were less frequently represented, appearing in four trials. Psychosocial and Rehabilitation components were interpreted as mainly representing the technical-therapeutic core of interventions, while Complementary components were interpreted as supporting the operational infrastructure of care, including assessment, planning, coordination, monitoring, referral, training, documentation, and continuity. **Conclusions:** This framework-guided secondary analysis suggests broad but uneven coverage of WHO long-term care domains across the included trials. Future trials should more explicitly align targeted needs, delivered components, and outcome assessment, including social, caregiver, palliative, continuity, and person-centred care experience outcomes.

## 1. Introduction

Population ageing and the growing prevalence of multimorbidity have increased the complexity of care needs among older people [1,2]. Multimorbidity is associated with a higher risk of care dependence, especially when physical, mental, and cognitive conditions co-occur, and complex multimorbidity is associated with greater need for assistance with instrumental activities of daily living [3,4]. In turn, functional disability is a central determinant of long-term care (LTC) needs and use, including formal and informal support [5]. In this context, LTC has become a priority for health and social support systems seeking to respond to prolonged, fluctuating, and cumulative trajectories of declining intrinsic capacity and functioning [1,2,6].

According to the World Health Organization (WHO), long-term care comprises health services, personal care, and social support, delivered continuously or intermittently, with the aim of maintaining, recovering, or optimising the functioning, dignity, autonomy, and rights of older people [2]. This definition moves beyond a narrow understanding of LTC as institutionalisation or biomedical disease management. Instead, it reinforces the need for person-centred, integrated models that are adapted to the resources of each context and capable of articulating clinical, functional, social, family-related, and continuity needs [2,7,8].

Person-centred interventions are particularly relevant in this context because they seek to address the clinical, functional, psychological, and social complexity of older people with multimorbidity [9,10]. In this regard, interventions based on comprehensive assessment, continuity of care, caregiver integration, and multidisciplinary teams may reduce some indicators of hospital use, although evidence remains more limited for other healthcare utilisation outcomes [11].

The parent review identified and standardised person-centred interventions in long-term care, distinguishing Psychosocial interventions, Rehabilitation interventions, and Complementary interventions according to their technical nature, and summarising the reported health gains [10]. This classification helped organise a heterogeneous field of interventions by clarifying what types of interventions had been evaluated. However, it did not examine how the delivered or reported components of these interventions corresponded to the long-term care needs and facilitating factors proposed by the World Health Organization [2].

This distinction is relevant because technical classifications and needs-oriented frameworks answer related but different questions. A technical classification mainly addresses what type of intervention was delivered, whereas a needs-oriented framework helps clarify which care needs were addressed and which delivery conditions supported care. This is consistent with integrated and person-centred approaches to older people with complex needs, which emphasise coordination, assessment, care planning, interprofessional collaboration, education, self-management support, and adaptation to local care contexts [8,9]. The World Health Organization long-term care framework adds a specific needs-oriented structure by organising long-term care into three core groups of needs—health care needs, palliative care needs, and social care and support needs—and two facilitating factors—person-centred integrated care and education and training [2].

The present study therefore does not update the parent review, reassess intervention efficacy, or replace the original technical classification. Instead, it re-examines the same set of randomised trials through a needs-oriented lens, consistent with framework-guided approaches that use a prior conceptual structure to organise, compare, and interpret evidence transparently [12,13,14]. Accordingly, this study aimed to conduct a framework-guided secondary analysis of the 18 randomised controlled trials included in the parent review, mapping the delivered or reported components of person-centred interventions onto the domains of the World Health Organization long-term care framework [10]. Thus, the research question was: which long-term care needs and facilitating factors are addressed by the delivered or reported components of person-centred interventions for older people with multimorbidity in long-term care or settings relevant to long-term care?

## 2. Materials and Methods

### 2.1. Study Design

This study was a framework-guided secondary analysis of the 18 randomised controlled trials included in the systematic review by Lista et al. [10]. The parent review identified and classified person-centred psychosocial and rehabilitation interventions targeting older people with multimorbidity in long-term care or settings relevant to long-term care.

The present analysis examined the same trials to answer the following research question: Which long-term care needs and facilitating factors are addressed by the delivered or reported components of the included interventions? To answer this question, the WHO package *Long-term care for older people: package for universal health coverage* [2] was used as the organising framework.

The analytical approach was informed by the matrix logic of the Framework Method [12] and by principles of best fit framework synthesis [13,14], particularly the use of a prior framework and transparent documentation of coding decisions. Trials were organised as rows in the working matrix and framework domains as columns. The primary unit of synthesis was the trial; intervention components were used as nested descriptive units to justify domain mapping.

### 2.2. Included Studies

The studies analysed were the 18 randomised controlled trials included in the systematic review by Lista et al. [10], comprising 9132 participants. The parent review included older people aged 65 years or over, with a baseline mean age of at least 70 years, with multimorbidity, in long-term care or settings relevant to long-term care. Multimorbidity was defined as the presence of two or more chronic conditions.

The included trials also required evidence of functional impairment or dependence in basic or instrumental activities of daily living. Trials comparing person-centred psychosocial and/or rehabilitation interventions with usual care were eligible.

The parent review covered a range of LTC contexts, including home care, community care, day centres, residential care homes and nursing homes, assisted living facilities, and primary or community care settings when addressing prolonged needs for functional support and chronic disease management. This delimitation was maintained in the present analysis. No additional literature search, screening, eligibility assessment, or exclusion of trials was conducted for the present secondary analysis. All 18 randomised controlled trials included in the parent review were retained and re-examined using the World Health Organization long-term care framework. The characteristics of the included trials, populations, settings, evaluated interventions, and comparators are summarised descriptively in the Section 3.

### 2.3. Organising Framework

The organising framework was the WHO package *Long-term care for older people: package for universal health coverage* [2]. LTC is understood as the set of activities ensuring that people with continued and significant intrinsic capacity loss can maintain a level of functioning consistent with their basic rights, fundamental freedoms, and human dignity [6]. LTC addresses health, personal care, and social needs, and may be provided on a continuous or intermittent basis over prolonged periods to people with substantially reduced intrinsic capacity who require support to maintain, recover, or optimise their functioning [2].

The package organises LTC into three core groups of needs: health care needs, palliative care needs, and social care and support needs. It also identifies two cross-cutting facilitating factors: person-centred integrated care and education and training. These five elements were used as fixed coding domains.

The framework was used to map the LTC needs addressed by the delivered or reported intervention components. It was not used to reclassify the technical nature of interventions as described in the parent review, nor to create new intervention categories. Subitems described in the WHO package, such as mobility, falls, polypharmacy, activities of daily living, social participation, and caregiver support, were used only as reference points for interpreting each domain. The main analysis remained at the level of the five framework domains, without creating additional subcategories.

### 2.4. Data Sources and Extracted Material

The analysis drew on trial-level data extracted from the studies included in the parent review. Primary trial reports, Supplementary Materials, final reports, and process evaluations were considered whenever they contained relevant information on delivered components, mode of delivery, professionals involved, resources, duration, adaptation, fidelity, outcomes, and reported results.

Trial protocols, registries, and planning documents were used only to clarify contextual information or to identify elements that were planned but not reported as delivered or evaluated. These elements were recorded separately as planned-only and did not contribute to the main mapping.

The extracted material was organised in a working matrix containing information on study characteristics, evaluated interventions, delivered or reported components, WHO framework domains, assessed outcomes, and coding decisions. Coding decisions requiring greater interpretation were documented in the decision log of the mapping workbook.

### 2.5. Framework Mapping Procedure

Mapping was conducted deductively from the five domains defined in the WHO long-term care package [2]. For each trial, intervention components described as delivered, used, implemented, or reported as part of the intervention were identified and compared against the operational definitions of each framework domain.

Because the parent review included only trials of person-centred psychosocial and/or rehabilitation interventions [10], person-centred integrated care was maintained as a fixed facilitating factor of the WHO framework, not as a category created post hoc in this analysis. Its coding remained contingent on the presence of delivered or reported components, such as patient narrative, shared goal setting, individualised care plan, care coordination, structured follow-up, or shared documentation.

Each domain was coded as present or absent at trial level. To code a domain as present, at least one intervention component described as delivered, used, implemented, or reported as part of the intervention was required. Domains were not treated as mutually exclusive, since a single intervention could simultaneously address clinical, social, palliative, or integrated care needs. When insufficient evidence was available to sustain domain mapping, the domain was coded as absent.

Coding was based on concrete actions described in the trial reports, not on labels assigned by authors to their interventions. Assessed outcomes, quality indicators, general intentions, and broad care model designations were not, in themselves, considered sufficient to activate a domain. Broad care models were decomposed into their explicitly described subcomponents, such as comprehensive assessment, individualised plan, structured follow-up, symptom management, medication review, functional training, caregiver support, referral, or service coordination, and coding was conducted at the level of those subcomponents.

Comprehensive assessment was coded by default under person-centred integrated care, consistent with multidomain approaches addressing the needs of older people [2,15]. It contributed to other domains only when the trial described a concrete subsequent action in that domain, such as symptom management, functional training, caregiver support, advance care planning, or referral to social services.

When information was ambiguous, a conservative decision was made and recorded in the decision log. Components described only in protocols, registries, or planning documents, without evidence of delivery or evaluation, were classified as planned-only and excluded from the main mapping.

### 2.6. Decision Log and Second-Reviewer Check

Coding decisions requiring greater interpretation were recorded in the decision log of the mapping workbook. Initial mapping was conducted by the first author using the predefined operational definitions, codebook, and working matrix. To standardise interpretive decisions, components were coded only when they were described as delivered, used, implemented, or reported as part of the intervention. Cases requiring greater judgement were flagged during coding and recorded in the decision log, together with the rationale for the final decision.

Second-reviewer verification focused on higher-judgement coding decisions, including borderline cases related to social care and support needs, palliative care needs, education and training, and care setting classification. The aim was to confirm consistency in the application of coding rules, not to repeat full data extraction. The second reviewer agreed with all decisions recorded in the decision log. During the broader mapping review, two material revisions were identified and incorporated into the final workbook: education and training for Nielsen et al. [16], and care setting classification for Counsell et al. [17]. This step reinforced the traceability of decisions and the internal consistency of the mapping.

### 2.7. Data Synthesis

Synthesis was descriptive and organised at trial level. For each WHO framework domain, the absolute number and percentage of trials mapped to that domain were calculated. As domains were not mutually exclusive, the same trial could contribute to more than one domain.

Results were organised in the Section 3 to describe the included trials, populations, settings, evaluated interventions, comparators, framework-domain mapping, and outcome areas assessed across trials.

Outcomes were grouped by construct, allowing comparison of evaluated areas across trials using heterogeneous measures, while detailed instruments were retained in the mapping workbook. No effects were recalculated, no additional statistical analyses were conducted, and no new quantitative synthesis of trial results was performed. Interpretation focused on the coverage of LTC needs by intervention components, not on reassessing the clinical efficacy of the interventions.

As a descriptive contextual analysis, domain mapping was also summarised by care setting and LTC relevance to examine whether patterns of WHO domain coverage differed across delivery contexts.

## 3. Results

### 3.1. Characteristics of Included Trials and Interventions

Table 1 presents the characteristics of the 18 randomised controlled trials included in this secondary analysis. Overall, the studies included 9132 participants from the systematic review by Lista et al. [10].

Six studies were classified as home-based long-term care, three as community-based long-term care, two as facility-based long-term care, and seven as health-care settings relevant to long-term care. This latter category included studies conducted in primary care, chronic disease programmes, post-acute rehabilitation, telehealth, and specialised health programmes.

The evaluated interventions were mostly multicomponent. All included Psychosocial and Complementary components; four also included Rehabilitation components. Intervention duration ranged from one or two sessions to programmes lasting 24 months. Comparators were generally described as usual care, although some studies used active or enhanced comparators, such as collaborative dementia care, educational materials, or usual reactive consultations.

### 3.2. Mapping to the WHO Long-Term Care Framework

Table 2 presents the study-by-study mapping of delivered or reported components to the domains of the WHO framework.

**Figure 1:**
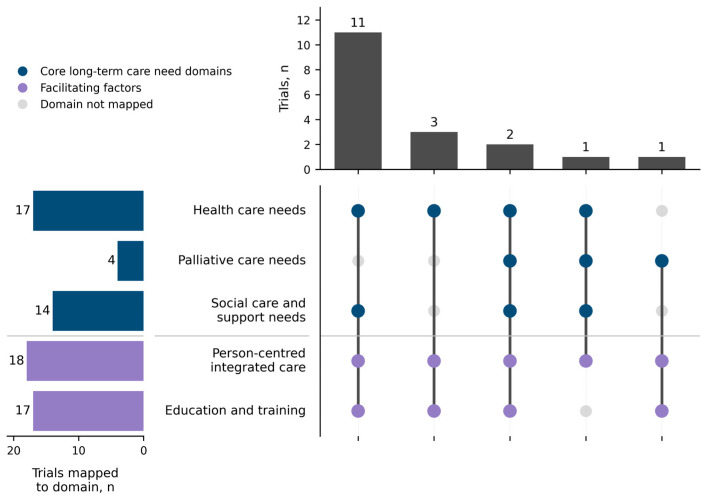
Patterns of World Health Organization long-term care domain coverage across included trials. Note. Bars show the number of trials mapped to each observed combination of domains, and horizontal bars show the total number of trials mapped to each domain. Filled circles indicate mapped domains and grey circles indicate domains not mapped. Domains were not mutually exclusive and are ordered according to the World Health Organization framework.

Domain coverage ranged from three to five domains per trial. Two trials were mapped to all five framework domains. Twelve trials were mapped to four domains, and four trials to three domains. The most frequent pattern was the combination of health care needs, social care and support needs, person-centred integrated care, and education and training, without palliative care needs, observed in 11 trials. Figure 1 presents the distribution of WHO domain coverage patterns across the included trials.

Health care needs were identified in 17 trials (94.4%). Components supporting this mapping included clinical assessment or monitoring, symptom management, medication review, chronic disease management, functional training, structured exercise, physical, cognitive, or occupational rehabilitation, clinical education, and referral to health care services.

Social care and support needs were identified in 14 trials (77.8%). Components supporting this mapping included support with activities of daily living or instrumental activities, caregiver support, caregiver training, linkage to social or community resources, social participation, meaningful activities, assistive products, environmental adaptation, and navigation across health and social services. Four trials were not mapped to this domain.

Palliative care needs were identified in four trials (22.2%). Components supporting this mapping included specialist palliative care, symptom management in a palliative context, advance care planning, advance directives, surrogate decision-maker appointment, planning for clinical deterioration, and care oriented towards comfort or end of life.

Person-centred integrated care was identified in all 18 trials (100.0%). Components supporting this mapping included comprehensive assessment, patient narrative, shared goal setting, individualised care plan, care coordination, case management, structured follow-up, reassessment, and shared documentation.

Education and training were identified in 17 trials (94.4%). Components supporting this mapping included training, supervision, or capacity-building for professionals, facilitators, intervention teams, workers involved in care delivery, or informal caregivers.

When mapping was summarised by care setting/LTC relevance, health care needs and person-centred integrated care were the most transversal domains across contexts. Social care and support needs were more frequent in home-based long-term care and community-based long-term care contexts. Palliative care needs appeared in trials classified as facility-based long-term care and home-based long-term care, with these mappings appearing proportionally more often in facility-based trials. Palliative care needs were not identified in trials classified as health-care settings relevant to long-term care. The full distribution by context is presented in Supplementary Table S1. Overall, the counts and percentages reported in this section should be interpreted as descriptive study-level mapping frequencies across the 18 included trials. They summarise how delivered or reported intervention components were mapped to the World Health Organization framework domains and care settings, and do not represent effect estimates, population prevalence, or statistical comparisons between domains or settings.

### 3.3. Outcome Domains Assessed in Trials Mapped to WHO Long-Term Care Domains

Table 3 summarises the outcome domains assessed in trials mapped to each domain of the WHO framework. Outcomes were grouped by construct rather than by instrument.

In trials mapped to health care needs, the assessed outcome domains included quality of life, health-related quality of life, symptoms, functioning, activities of daily living, participation, depressive symptoms, anxiety, cognition, self-management, self-efficacy, patient activation, service use, hospitalisation, mortality, costs, and safety.

In trials mapped to social care and support needs, the assessed outcome domains included functioning, activities of daily living, participation, service use, social support, loneliness, caregiver-related outcomes, and qualitative experience.

In trials mapped to palliative care needs, the assessed outcome domains included symptoms in a palliative context, advance care planning, advance directive completion, surrogate decision-maker appointment, preferred place of death, quality of death, mortality or survival, service use, caregiver burden, and qualitative perceptions.

In trials mapped to person-centred integrated care, the assessed outcome domains included satisfaction with care, care experience, perceived quality, continuity, perceived integration, quality indicators, fidelity, dose, intervention use, and qualitative experience.

In trials mapped to education and training, the assessed outcome domains included fidelity, delivery quality, dose, professional experience, staff confidence, death literacy, caregiver competence, supporter competence, and implementation-related outcomes.

## 4. Discussion

### 4.1. Reframing Person-Centred Interventions Through Long-Term Care Needs

Understanding which care needs are effectively covered by person-centred interventions remains challenging in long-term care (LTC), given the heterogeneity of components, contexts, and outcomes across existing interventions [11]. In the parent review, this heterogeneity was organised according to the technical nature of interventions, distinguishing Psychosocial, Rehabilitation, and Complementary components [10]. This classification helped clarify what type of intervention was evaluated and what health gains were reported, but it did not explain how delivered components aligned with the long-term care needs framework proposed by the World Health Organization (WHO), which organises essential care across health and social sectors within formal and integrated systems oriented towards universal coverage [1,2].

The present secondary analysis revisits the same trials from a different perspective. Rather than asking what technical type of intervention was evaluated, it seeks to understand which LTC needs are addressed by the components effectively delivered or reported. This reading draws on the analytical notion of care functions, that is, the role played by components within the care process. This reading does not constitute a new taxonomy, nor does it reclassify the parent review. Rather, it synthesises how components operate in LTC. This approach is consistent with the literature on complex interventions, which recommends considering context, implementation, mechanisms, and the contribution of components beyond effect estimation [34,35].

**Figure 2:**
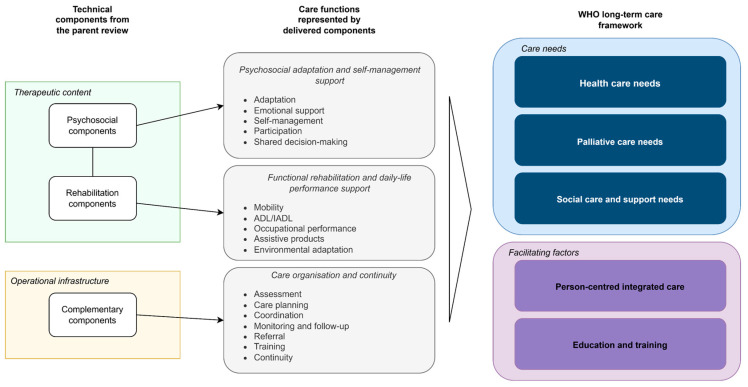
Conceptual synthesis of technical intervention components and WHO long-term care domains. Note. Technical components from the parent review were interpreted according to their functional role in long-term care and mapped to the WHO long-term care domains. Arrows indicate conceptual links, not one-to-one equivalence.

When read through this lens, Psychosocial and Rehabilitation components mainly represent the technical-therapeutic core of the intervention. The former address psychosocial dimensions of the experience of chronic illness or dependence, including psychosocial adaptation, emotional support, self-management, participation, and shared decision-making. The latter address dimensions related to functioning and occupational performance, including mobility, activities of daily living and instrumental activities, assistive products, and environmental adaptation. Both have greater therapeutic density in the original classification, although they may act through different pathways and respond to more than one needs domain. In contrast, Complementary components, classified as having lower specialised therapeutic density, emerged in this analysis as the operational infrastructure of care, supporting processes of assessment, planning, coordination, monitoring, referral, training, documentation, and continuity. Figure 2 represents this conceptual interpretation, linking the components identified in the parent review to their functions within the care process before mapping them onto the WHO framework domains.

The results showed broad but uneven coverage of the framework domains. Health care needs, social care and support needs, person-centred integrated care, and education and training were frequently represented, whereas palliative care needs were less frequent. This distribution informs the three interpretive readings developed in the following subsections: the reinterpretation of Complementary components as the operational infrastructure of care, the lower visibility of palliative care needs, and the alignment between delivered components and assessed outcomes.

### 4.2. Complementary Components as Operational Infrastructure of Care

The reinterpretation of Complementary components constitutes a central conceptual contribution of this secondary analysis. In the parent review, these components were distinguished from Psychosocial and Rehabilitation components because they had lower specialised technical-therapeutic density or weaker anchoring in formal intervention models [10]. However, when read through the WHO framework, their relevance extends beyond therapeutic density. These components make visible an operational layer of care that organises, sustains, and adjusts intervention delivery over time. We conceptualise this role as the operational infrastructure of care.

The expression operational infrastructure of care does not correspond to a formal category in the parent review. It is an analytical conceptualisation of this study, used to describe enabling functions such as assessment, planning, coordination, monitoring, referral, documentation, training, and continuity. This operational layer helps explain how components classified as Complementary could support several framework domains, especially person-centred integrated care and education and training, while also contributing to health care needs, social care and support needs, or palliative care needs when linked to concrete actions in those domains. Their contribution lies less in adding new technical-therapeutic content than in making such content actionable within the person’s care pathway.

Several included trials illustrate this pattern. In Counsell et al. [17] and Spoorenberg et al. [33], therapeutic content was delivered within formal case-management or integrated care models. In Forbat et al. [25], Needs Rounds functioned as a structured mechanism for shared clinical decision-making and linkage to specialist resources in residential care. In Fisher et al. [24], operational components supported a multiprofessional home-based intervention through assessment, planning, follow-up, and referral. These examples suggest that Complementary components helped integrate psychosocial or rehabilitation content into continuous and adjustable care processes.

The literature on integrated and multicomponent models for older people with complex needs converges in this direction. Bayly et al. [9] identified interprofessional collaboration, continuous assessment, active participation of the person, education of the person and family, and self-management as common elements. Wodchis et al. [8], in a comparative analysis of seven international integrated care programmes, reinforced the importance of coordination, integration between health and social support, case management, and adaptation to local contexts. Taken together, these contributions support the interpretation of Complementary components as enabling functions that allow therapeutic content to be embedded in integrated, person-centred care.

This reading aligns with the logic of the WHO long-term care package, which frames LTC not only as a response to clinical, palliative, and social needs, but also as a process requiring enabling conditions for organised and sustained delivery [2]. In the analysed trials, care planning, case management, follow-up, shared documentation, training, and supervision gave concrete form to person-centred integrated care and education and training. When these operational functions included medication review, referral, caregiver support, or advance care planning, they also contributed to specific clinical, social, or palliative domains.

The term Complementary may therefore invite a reductive interpretation if read only through therapeutic density. The label reflects the technical function assigned in the parent review, not the structural value of these components in LTC. At system level, this is relevant because formal LTC models depend on the capacity to integrate essential interventions across health and social sectors, within accessible and adequately supported care systems [1,2]. For nursing practice, this argument is also important. Care coordination derives from the person’s clinical situation and the complexity of their needs, rather than from disciplinary exclusivity [7]. In contexts of multimorbidity and dependence, nursing may occupy a strategic position in multidimensional assessment, education, monitoring, and articulation between the person, family, team, and services [2,7]. In this analysis, Complementary components helped transform technical interventions into integrated, supported, and adjustable care processes over time.

### 4.3. Making Palliative Care Needs Explicit in LTC

The lower representation of palliative care needs was the clearest asymmetry in the mapping, with only 4 of the 18 trials coded in this domain. This finding warrants cautious interpretation. The WHO framework served as an analytical lens to identify represented needs, rather than as a normative checklist to evaluate domain coverage. Therefore, the absence of mapping to this domain does not imply methodological incompleteness in the remaining trials.

A substantive distinction is also necessary. In this analysis, the presence of chronic disease, frailty, multimorbidity, or functional dependence did not, by itself, classify an intervention as palliative. Although the WHO long-term care package integrates health, palliative, and social support needs within a person-centred continuum [2], palliative care needs were coded only when trials reported explicit components. These included specialist palliative care, advance care planning, advance directives, surrogate decision-maker appointment, planning for clinical deterioration, end-of-life care, or symptom management in a palliative context. This conservative strategy prevented conflating population vulnerability with the actual delivery of palliative care.

The scope of the parent review, which targeted person-centred Psychosocial and Rehabilitation interventions, may partly explain this pattern [10]. However, it may also indicate that palliative needs were less often translated into explicit, reportable, and evaluable components within the analysed trials. Person-centredness was predominantly operationalised through assessment, planning, coordination, self-management, psychosocial support, and rehabilitation. By contrast, suffering, comfort, end-of-life preferences, advance decisions, and family support in a palliative context were less prominent. This distinction is relevant because palliative care needs in older people with multimorbidity encompass physical, psychosocial, family, informational, and organisational dimensions that may remain under-recognised unless explicitly embedded in intervention design [36]. This is particularly relevant in LTC, where care often responds to prolonged and progressive trajectories of intrinsic capacity loss, while aiming to preserve functioning, dignity, autonomy, and fundamental rights [6].

Consequently, the lower representation of palliative care needs should be interpreted as limited operationalisation within delivered components, not as evidence that such needs were absent in the populations studied. Palliative care, rehabilitation, psychosocial support, and care coordination may coexist in older people with advanced or progressive conditions. Bayly et al. [9] highlighted this overlap in geriatric and palliative care models, where care may span the continuum from preventing functional decline to end-of-life care. This reinforces the need to distinguish between populations with potential palliative needs and the explicit integration of palliative components within intervention design and reporting.

### 4.4. Aligning Intervention Components and Outcome Assessment

The analysis of outcome domains also shows that the breadth of delivered components was not always matched by equally comprehensive outcome assessment. As shown in Table 3, clinical, functional, psychological, quality-of-life, and service-use outcomes appeared across several mapped domains, whereas caregiver, social, continuity, training-related, and person-centred care experience outcomes were assessed less consistently. Interventions that included caregiver support or linkage to community resources did not always assess caregiver burden, social support, or access to services. Similarly, interventions that operationalised advance care planning did not always capture preferences, advance directive completion, or place of death.

This misalignment is not trivial. LTC is not limited to disease management. Its purpose is to support older people in maintaining functioning, autonomy, participation, dignity, and quality of life across trajectories that are often prolonged and unstable [1,2]. The multidimensional conceptualisation of quality of life includes physical health, psychological state, independence, social relationships, environment, and spiritual or existential dimensions [37]. Assessments centred too narrowly on physical health may therefore miss dimensions that are critical for older people in home-based and community-based contexts, such as autonomy, environment, social relationships, and material well-being [38]. This issue is particularly sensitive in models that integrate palliative needs, where the selection of person-centred outcomes is essential to capture preferences, care experience, quality of life, and alignment between intervention and the person’s needs [39].

Interpretive caution is nevertheless required. The inconsistent assessment of social, palliative, family-related, training-related, or continuity outcomes does not, in itself, represent a methodological weakness of the primary trials. Studies selected outcomes that were coherent with their specific aims. Moreover, evaluating integrated and multicomponent interventions in older people with complex needs is methodologically demanding, particularly when trying to understand how interacting components, implementation processes, and context shape outcomes [34,35].

### 4.5. Implications for Long-Term Care Models and Nursing

The findings support a different way of thinking about LTC models. Rather than starting from techniques, professions, or places of care delivery, the proposed reading organises the discussion around needs and care functions. This perspective has practical consequences for model design, governance, and the professional profiles that sustain them.

At the organisational level, formal LTC models integrated across the health and social sectors need to make explicit how they articulate clinical, functional, social, family-related, and continuity needs. The WHO package offers an organising framework for this articulation, but its implementation depends on local decisions about funding, regulation, human resources, and intersectoral governance [1,2]. The capacity-building of multidisciplinary teams and informal caregivers emerges, within this framework, as a structural condition for model sustainability, not as an optional layer.

For professional practice, the argument points to a shared space of contribution across disciplines, particularly in community, home-based, rehabilitation, and palliative contexts. Given its proximity to multidimensional assessment, monitoring, education, and articulation between the person, family, team, and services, nursing may occupy a strategic position in sustaining this operational infrastructure [2,7]. The consolidation of LTC models will ultimately depend on the ability to integrate therapeutic content, operational functions, and the person’s real needs into a coherent care pathway.

From a research perspective, future long-term care trials should move from broad person-centred labels to a prospective specification of targeted needs, delivered components, responsible actors, implementation processes, and assessed outcomes. The WHO long-term care framework can support this work at protocol stage by helping research teams define which domains are central to the intervention and which outcomes are needed to capture them [2]. A trial focused on rehabilitation may legitimately prioritise functioning and participation outcomes, whereas a trial involving advanced multimorbidity, frailty, care transitions, or progressive decline should consider whether palliative, caregiver, continuity, and social support outcomes are also required. Clear reporting of dose, fidelity, adaptation, context, and delivery mechanisms would further strengthen interpretation and transferability across long-term care systems [34,35]. For policymakers, this alignment offers a practical route for designing accountable care pathways in which clinical, social, palliative, educational, and person-centred care functions are specified rather than assumed [2].

### 4.6. Strengths and Limitations

The study design has several strengths. The 18 trials were drawn from a published systematic review [10], ensuring a predefined and methodologically documented selection base. The framework used is international and has direct applicability to the planning of LTC models [2]. The operational rules prioritised delivered or reported components, excluding planned-only elements from the main mapping. The unit of synthesis was the trial, with interpretive decisions recorded in a decision log and targeted verification by a second reviewer. The codebook, mapping matrix, and decision log were organised as supporting materials to strengthen the traceability and reproducibility of the mapping.

Several limitations should also be acknowledged. The mapping depended on the reporting quality of the original trials, meaning that delivered but poorly described components may not have been captured. Consequently, less formalised components, such as informal professional training, ad hoc caregiver guidance, practical social support, or contextual adaptations during delivery, may have been underestimated when they occurred in practice but were not sufficiently described in the published reports. This limitation is particularly relevant in complex interventions, where incomplete description of components, materials, procedures, dose, adaptation, or fidelity can hinder interpretation and replication [40]. The universal coverage of person-centred integrated care should also be interpreted in light of the parent review inclusion criteria, which selected person-centred psychosocial or rehabilitation interventions. Rather than an emergent finding, this result confirms that the interventions retained a person-centred architecture when reanalysed through the WHO framework for LTC. The more informative contrast therefore lies in the uneven coverage of the remaining domains, especially the lower representation of palliative care needs.

In addition, study-level coding identified the presence or absence of domains, but did not quantify the intensity, dose, quality, or relative centrality of each component within the intervention. Two trials mapped to the same domain may therefore have operationalised that domain with different levels of depth. Finally, this analysis did not reassess risk of bias, recalculate effects, or conduct a new quantitative synthesis of trial results. The findings should therefore be interpreted as a mapping of care functions and needs, rather than as a new assessment of the clinical impact of the interventions.

## 5. Conclusions

This framework-guided secondary analysis suggests that the person-centred interventions included in the parent review can be interpreted through a needs-oriented LTC lens. Delivered or reported components were broadly mapped to health care needs, social care and support needs, person-centred integrated care, and education and training, but less frequently to palliative care needs.

In this analysis, Psychosocial and Rehabilitation components were interpreted as mainly representing the technical-therapeutic core of the interventions, whereas Complementary components were interpreted as supporting the operational infrastructure of care. Future LTC studies should more explicitly specify the relationship between targeted needs, delivered components, implementation processes, and assessed outcomes, including social, caregiver, palliative, continuity, and person-centred care experience dimensions.

## Figures and Tables

**Table 1:** Characteristics of included randomised trials and interventions.

Study	Country	Population/Condition	Sample	Care Setting/LTC Relevance	Intervention Duration	Intervention Evaluated	Comparator	Core and Complementary Components Represented
**Ali et al.** [18]	Sweden	Chronic obstructive pulmonary disease or heart failure	n = 222	LTC-relevant health settings	6 months	Person-centred digital platform and structured telephone support	Usual care	Psychosocial + Complementary
**Callahan et al.** [19]	United States	Alzheimer’s disease	n = 180	Home-based long-term care	24 months	Home-based occupational therapy added to collaborative dementia care	Collaborative dementia care	Psychosocial + Rehabilitation + Complementary
**Chen et al.** [20]	Taiwan	High-need community-dwelling older people	n = 145	Community-based long-term care	6 months	High-Need Community-Dwelling Older Adults Care Delivery Model	Usual care	Psychosocial + Complementary
**Connor et al.** [21]	United States	Parkinson’s disease	n = 328	LTC-relevant health settings	18 months	CHAPS nurse-led Parkinson’s disease care management	Usual care + educational handout	Psychosocial + Complementary
**Counsell et al.** [17]	United States	Low-income older people with multimorbidity and geriatric syndromes	n = 951	Home-based long-term care	24 months	GRACE geriatric care management	Usual care	Psychosocial + Complementary
**Dalal et al.** [22]	United Kingdom	Heart failure with reduced ejection fraction	n = 216	LTC-relevant health settings	12 weeks	REACH-HF home-based cardiac rehabilitation and self-care	Usual care	Psychosocial + Rehabilitation + Complementary
**Evans et al.** [23]	United Kingdom	Frail older people with chronic non-cancer conditions and palliative/supportive care needs	n = 50	Home-based long-term care	12 weeks	SIPScare integrated palliative and supportive care	Usual care	Psychosocial + Complementary
**Fisher et al.** [24]	Canada	Older people with multimorbidity newly referred to home care	n = 59	Home-based long-term care	6 months	Interprofessional home-care self-management intervention	Usual home care	Psychosocial + Complementary
**Forbat et al.** [25]	Australia	Residential aged care residents	n = 1700 residents	Facility-based long-term care	Variable, 6–14.5 months	“Needs Rounds” specialist palliative care in residential care homes	Usual reactive consultations	Psychosocial + Complementary
**Fors et al.** [26]	Sweden	Chronic obstructive pulmonary disease or heart failure	n = 221	LTC-relevant health settings	6 months	Person-centred telephone support	Usual care	Psychosocial + Complementary
**Fu et al.** [27]	New Zealand	Community-living post-stroke adults	n = 400	LTC-relevant health settings	1–2 sessions over 6 weeks	“Take Charge” post-stroke self-management intervention	Educational materials + usual care	Psychosocial + Complementary
**Gilbody et al.** [28]	United Kingdom	Older people with subthreshold depression	n = 705	LTC-relevant health settings	7–8 weeks	CASPER collaborative care for subthreshold depression	Usual care	Psychosocial + Complementary
**Melis et al.** [29]	Netherlands	Frail or vulnerable community-dwelling older people	n = 151	Home-based long-term care	3 months	Dutch EASYcare geriatric home-visit programme	Usual care	Psychosocial + Rehabilitation + Complementary
**Mountain et al.** [30]	United Kingdom	People with mild dementia	n = 480	Community-based long-term care	12 weeks	“Journeying through Dementia” psychosocial intervention	Usual care	Psychosocial + Complementary
**Nielsen et al.** [16]	Denmark	Older people with occupational performance problems using or seeking home care	n = 119	Home-based long-term care	11 weeks	Intensive client-centred occupational therapy	Usual practice	Psychosocial + Rehabilitation + Complementary
**Overbeek et al.** [31]	Netherlands	Frail older adults in residential care or home care	n = 201	Facility-based long-term care	Mean 47 days	“Respecting Choices” advance care planning	Usual care	Psychosocial + Complementary
**Salisbury et al.** [32]	United Kingdom	Adults with multimorbidity	n = 1546	LTC-relevant health settings	15 months	3D primary care multimorbidity review	Usual care	Psychosocial + Complementary
**Spoorenberg et al.** [33]	Netherlands	Community-living older people aged 75 years or over	n = 1456	Community-based long-term care	12 months	Embrace integrated care model	Usual care	Psychosocial + Complementary

Note. LTC = long-term care. LTC-relevant health settings refer to health-care settings relevant to long-term care, including primary care, chronic disease programmes, post-acute rehabilitation, telehealth or specialised health programmes addressing sustained clinical, functional or supportive needs. In the parent review, core interventions comprised Psychosocial and Rehabilitation components. Complementary components referred to supporting care-process components used to operationalise, integrate, coordinate or sustain intervention delivery. Quotation marks identify formal intervention names reported by the trial authors.

**Table 2:** Mapping of intervention packages to the WHO long-term care framework.

Study	Health Care Needs	Palliative Care Needs	Social Care and Support Needs	Person-Centred Integrated Care	Education and Training
**Ali et al.** [18]	Symptom self-monitoring; clinical information on COPD/CHF; telephone support for clinical self-management	—	—	Cocreated health plan; patient narrative; structured telephone follow-up; digital documentation	Professional training in remote person-centred communication; fidelity meetings/review
**Callahan et al.** [19]	Home-based occupational therapy; ADL/IADL training; mobility/transfer training; exercise; cognitive strategies	—	Caregiver training; community resources; safe return programme; caregiver support group; home modification	Home assessment; individualised care plan; goal setting with dyad; dementia care management; coordination with primary care	Therapist training; caregiver education/training
**Chen et al.** [20]	Linkage to home nursing, medical treatment, rehabilitation and telecare; monitoring/reassessment	—	Home care; day care; assistive products; benefits consultation; caregiver training; respite care; emotional support	Screening; comprehensive assessment; individualised care plan; care coordination; follow-up; plan update	Three-day care coordinator training; monthly case discussions
**Connor et al.** [21]	Structured assessment; symptom/falls/medication indicators; clinical referrals; hospital admission notification	—	Caregiver support packet; caregiver assessment questions; Community/VA social services	Nurse care management; structured assessment; problem-specific plans; action plans; follow-up; specialist huddles	Process education for nurse care managers; team meetings/support
**Counsell et al.** [17]	Geriatric protocols; medication management; falls, pain, incontinence, depression, sensory deficits, malnutrition and dementia management	Advance care planning; health care representative/living will	Social worker involvement; caregiver burden protocol; transport support; community services	In-home comprehensive assessment; individualised care plan; interdisciplinary team; primary care integration; monthly follow-up	Twelve weekly seminars for support team; interdisciplinary team meetings
**Dalal et al.** [22]	Home-based cardiac rehabilitation; exercise; walking; symptom, fluid and medication management; breathlessness management	—	Family and Friends Resource; caregiver as co-facilitator	Tailored facilitation; goal setting; Progress Tracker; review of progress	Three-day facilitator training; fidelity checklist/audio review
**Evans et al.** [23]	Symptom management; follow-up/monitoring; management of pain, breathlessness, constipation, anxiety and drowsiness	Specialist palliative/supportive care; advance care planning; palliative symptom management; serious illness support	Caregiver support; psychosocial/practical support; integration with social/community services	Multidimensional assessment; priorities/preferences; care plan; specialist coordination; integration with GP/community nurses	—
**Fisher et al.** [24]	Standardised screening; medication review; interprofessional clinical input; communication with family physicians	—	Home care; personal support workers; caregiver involvement; system navigation; access to health/social services	Patient-as-partner approach; individualised care plan; monthly case conferences; case management	Two-day professional training; implementation meetings; audit/feedback
**Forbat et al.** [25]	Symptom assessment/management; medication review; anticipatory medication; direct clinical work; external referrals	Palliative care Needs Rounds; advance care planning; surrogate decision-maker; planning for deterioration; end-of-life care	Case conferences with relatives/decision-makers; family involvement; external referrals where relevant	Monthly structured Needs Rounds; checklist; case conferences; GP/care-home/specialist coordination	Case-based staff education; site briefing; fidelity feedback
**Fors et al.** [26]	Person-centred telephone support for COPD/CHF; discussion of medication, symptoms, sleep and clinical self-management	—	—	Patient narrative; co-created mailed health plan; goals; structured follow-up and plan revision	Nurse training; fortnightly meetings; peer review of calls/documentation
**Fu et al.** [27]	Post-stroke self-directed rehabilitation; stroke prevention; physical needs; mobility; ADL; communication	—	My Support Network; finances; daily life activities; family/friend involvement, actively worked through in sessions	Workbook-guided non-directive sessions; identity reflection; personal goals; hopes/fears; intermediate steps	Facilitator training; ongoing facilitator support
**Gilbody et al.** [28]	Behavioural activation for depressive symptoms; symptom monitoring; active surveillance; suicide-risk protocol	—	Signposting to voluntary/statutory services; social activation; functional equivalence activities	Case manager; agreed goals; session-by-session monitoring; GP/specialist liaison; PC-MIS	Case manager training; manualisation; weekly supervision
**Melis et al.** [29]	Management of cognition, nutrition, behaviour, mood, mobility/falls and medication; referrals	—	Home care/day care/voluntary care; assistive products; caregiver advice; ADL/IADL-related support	EASYcare assessment; goal setting; integrated individualised plan; GP-geriatrician-nurse coordination; follow-up	Psychoeducation/advice to caregiver
**Mountain et al.** [30]	Memory strategies; physical health; activity; diet; anxiety, stress and sleep topics	—	Peer support; friendships; community connectedness; out-of-venue activities; daily living topics	Flexible topic selection; individual sessions; personal goals; strengths-based approach; meaningful activities	Facilitator training; weekly supervision; fidelity checklists
**Nielsen et al.** [16]	Client-centred occupational therapy targeting occupational performance and functionality	—	Occupations/activities in home and local community; participation-oriented performance goals	Client-centred assessment; individualised goals; tailored occupational intervention	Therapist workshops; regular meetings/workshops; skills development or supervision supporting intervention delivery
**Overbeek et al.** [31]	—	Facilitated advance care planning; advance directives; surrogate decision-maker appointment	—	Facilitated conversations; structured interview cards; documentation of preferences/directives	Nurse facilitator training/certification; supervision
**Salisbury et al.** [32]	Depression review; medication review; clinical review of multimorbidity and health dimensions	—	—	Six-monthly person-centred reviews; named clinician; written health plan; electronic template; specialist advice link	Clinical/administrative training; 3D champion; monthly feedback
**Spoorenberg et al.** [33]	Assessment, monitoring, prevention and health-related case management	—	Care/support plan; caregiver support; navigation; community/well-being organisation linkages	Risk stratification; GP-led Elderly Care Team; case management; goal setting; individual plan; shared electronic record	Intensive team training and coaching

Note. COPD = chronic obstructive pulmonary disease; CHF = chronic heart failure; ADL = activities of daily living; IADL = instrumental activities of daily living; GP = general practitioner; VA = Veterans Affairs; PC-MIS = Patient Case Management Information System; WHO = World Health Organization. “—" indicates that no delivered or reported component met the operational criteria for that domain. Entries summarise the delivered or reported components that supported domain mapping and are not intended to provide exhaustive intervention descriptions. Full extraction details, coding decisions, and supporting rationale are available in the framework mapping workbook and decision log.

**Table 3:** Outcome domains assessed in trials mapped to each WHO long-term care domain.

WHO Long-term Care Domain/Facilitating Factor	Trials Mapped, n (%)	Outcome Areas Assessed in Trials Mapped to This Domain	Interpretive Summary	Less Consistently Assessed Outcome Areas
**Health care needs**	17 (94.4)	■ Anxiety ■ Cognition ■ Costs ■ Depression ■ Functioning, daily activities and participation ■ Health-related quality of life ■ Hospitalisation ■ Mortality ■ Physical performance ■ Quality of life ■ Safety/adverse events ■ Self-efficacy ■ Self-management ■ Service use ■ Symptoms	Health care needs were the most consistently represented domain. Outcomes mainly captured clinical status, functioning-related outcomes, quality of life, mental health, and service use. Functioning and daily activities were treated as cross-cutting outcomes.	■ Safety outcomes ■ Patient activation ■ Self-management ■ Clinically meaningful functional change
**Palliative care needs**	4 (22.2)	■ Advance care planning ■ Advance directive completion ■ Caregiver burden ■ Mortality/survival ■ Palliative symptom burden ■ Preferred place of death ■ Quality of death ■ Qualitative perceptions ■ Service use ■ Surrogate decision-maker appointment	Palliative outcomes were mainly assessed in trials where palliative care or advance care planning formed a clear part of the delivered intervention. Palliative-related indicators alone were not considered sufficient to classify interventions as addressing palliative care needs.	■ Family experience ■ Symptom relief ■ Quality of dying ■ Implementation of care preferences ■ Person-centred palliative outcomes
**Social care and support needs**	14 (77.8)	■ Caregiver burden ■ Caregiver competence ■ Caregiver quality of life ■ Community participation ■ Functioning, daily activities and participation ■ Loneliness ■ Qualitative experience ■ Service use ■ Social support	Social care and support overlapped with daily life and participation outcomes. However, social/support components were not always matched by outcomes directly measuring social participation, support networks, caregiver experience, or access to community resources.	■ Social participation ■ Loneliness ■ Support networks ■ Transport/accessibility ■ Respite care ■ Caregiver outcomes
**Person-centred integrated care**	18 (100.0)	■ Care experience ■ Chronic care assessment ■ Continuity ■ Implementation/fidelity ■ Intervention use/dose ■ Joined-up care ■ Perceived quality of care ■ Qualitative experience ■ Quality-of-care indicators ■ Satisfaction with care	Person-centred integrated care was common as a design and delivery feature, usually through assessment, individualised planning, goal setting, follow-up, care coordination, or case management. Direct measurement of person-centred care experience was less consistent.	■ Goal attainment ■ Shared decision-making quality ■ Person-centred care experience ■ Continuity ■ Perceived integration
**Education and training**	17 (94.4)	■ Caregiver competence ■ Delivery quality ■ Fidelity/process outcomes ■ Intervention dose ■ Professional experience ■ Staff confidence/death literacy ■ Supporter competence ■ Training-related implementation outcomes	Education and training were frequently used to support intervention delivery, especially for professionals, facilitators, case managers, care-home staff, or caregivers. These components were usually reported as part of implementation rather than evaluated as outcomes.	■ Staff competence ■ Training uptake ■ Fidelity ■ Caregiver competence ■ Delivery quality

Note. WHO = World Health Organization. Counts refer to study-level mapping in Table 2. Outcome areas are reported as constructs, not instruments. Outcomes listed in each row were assessed across trials mapped to that domain, not in every individual trial. Percentages are descriptive mapping frequencies based on the 18 included trials.

## Data Availability

The data used in this secondary analysis were extracted from published randomised controlled trials included in the parent review. Supplementary Materials, including the analysis plan, codebook, final framework mapping workbook, decision log, and second-reviewer validation records, are available through the Open Science Framework: https://osf.io/w5cp7/ (accessed on 15 May 2026).

## Supplementary Materials

The following supporting information can be downloaded at: https://www.mdpi.com/article/10.3390/healthcare14121623/s1, Table S1: WHO long-term care domain coverage by care setting/LTC relevance.
