# Supplementary material for: Mapping Long-Term Care Needs in Person-Centred Interventions for Older People with Multimorbidity: A WHO Framework-Guided Secondary Analysis
Source: Healthcare (Basel). 2026 Jun 9;14(12):1623. doi: 10.3390/healthcare14121623 (PMC13299773; doi:10.3390/healthcare14121623)
Supplement: Supplementary file 1 [file healthcare-14-01623-s001.zip › healthcare-4350359-supplementary.pdf]

Supplementary Table S1. WHO long-term care domain coverage by care setting / LTC relevance.

Care setting / LTC relevance	Trials, n	Health care needs, n/N (%)	Palliative care needs, n/N (%)	Social care and support needs, n/N (%)	Person-centred integrated care, n/N (%)	Education and training, n/N (%)
Home-based long-term care	6	6/6 (100.0)	2/6 (33.3)	6/6 (100.0)	6/6 (100.0)	5/6 (83.3)
Community-based long-term care	3	3/3 (100.0)	0/3 (0.0)	3/3 (100.0)	3/3 (100.0)	3/3 (100.0)
Facility-based long-term care	2	1/2 (50.0)	2/2 (100.0)	1/2 (50.0)	2/2 (100.0)	2/2 (100.0)
LTC-relevant health settings	7	7/7 (100.0)	0/7 (0.0)	4/7 (57.1)	7/7 (100.0)	7/7 (100.0)
**Total**	**18**	**17/18 (94.4)**	**4/18 (22.2)**	**14/18 (77.8)**	**18/18 (100.0)**	**17/18 (94.4)**

Note. WHO = World Health Organization; LTC = long-term care. Values are n/N (%), where N is the number of trials within each care setting / LTC relevance category. Domains were not mutually exclusive; therefore, the same trial could contribute to more than one domain. Mapping was based on delivered or reported intervention components.
